# Pediatric Orbital Floor Reconstruction Using a Nonresorbable Polypropylene-Based Hernia Mesh: A Case Report

**DOI:** 10.7759/cureus.34326

**Published:** 2023-01-29

**Authors:** Gauri Sharma, Deepankar Shukla, Nitin D Bhola, Rozina Vishnani

**Affiliations:** 1 Oral and Maxillofacial Surgery, Sharad Pawar Dental College and Hospital, Datta Meghe Institute of Higher Education and Research, Wardha, IND

**Keywords:** orbital floor fracture, orbital fracture, pediatric trauma, white eyed, orbit

## Abstract

Orbital floor fracture is the most common type of orbit-related injury seen in pediatric age groups. It is also called a *white-eyed* blowout fracture when the usual signs of orbital fracture-periorbital edema, ecchymosis, and subconjunctival hemorrhage are absent. Several materials are used for orbital defect reconstruction. The most popular and widely used material is titanium mesh. We present a case of a 10-year-old boy with a *white-eyed* blowout fracture of the floor of the orbit of the left side. The patient had a history of trauma, after which he developed diplopia in the left eye. On examination, his upward gaze was restricted in the left eye, which was suggestive of inferior rectus muscle entrapment. The orbital floor reconstruction was done using a hernia mesh made up of nonresorbable polypropylene material. This case demonstrates the utility of nonresorbable materials in orbital defect reconstruction in pediatric patients. Further studies are needed to better understand the scope of polypropylene-based materials in the floor of orbit reconstruction and their benefits and shortcomings in the long run.

## Introduction

The term *white-eyed* blowout fracture was coined by Jordan et al. to describe fractures of the orbit in pediatric age groups, with the features of minimal or no signs of periorbital ecchymosis and enophthalmos, presence of double vision with restricted upward gaze, and radiological signs of minimal bone displacement [1]. Orbital floor fracture is one of the most common types of orbit-related injury seen in the pediatric age group. However, these kinds of fractures are mostly seen in children aged more than seven years due to pneumatization of maxillary sinuses, which happens after eight years of age [2].

Around 10% of cases of pediatric facial fractures are orbital fractures according to a review by the *National Trauma Databank* [3]. Frequently orbital fractures in children are accompanied by the oculocardiac reflex (OCR), also known as the Aschner phenomenon. The most commonly accepted theories for blowout fractures of the orbit are the *hydraulic theory*, *globe-to-wall theory*, and *buckling theory* [1,4].

Orbital floor fractures have a trapdoor-like effect. The inferior rectus muscle and other orbital contents such as connective tissue and fat can get herniated within the fracture line. The floor of the orbit reconstruction aims to release the soft tissues entrapped within the fracture line, restore restricted gaze, and correct orbital volume defects if present. Orbital floor repair can be done by autologous, allogenic, alloplastic, and xenogenic implants. The most commonly used titanium mesh is an example of an alloplastic implant. Polypropylene-based mesh is a nonresorbable alloplastic material routinely used in hernia surgeries. The rationale for using a lightweight porous material is to provide adequate support and integration to the adjacent areas [5].

Orbital defects have been repaired using semiabsorbable mesh. In this case, a nonresorbable polypropylene mesh is used for the floor-of-orbit defect reconstruction.

## Case presentation

**Figure 1:**
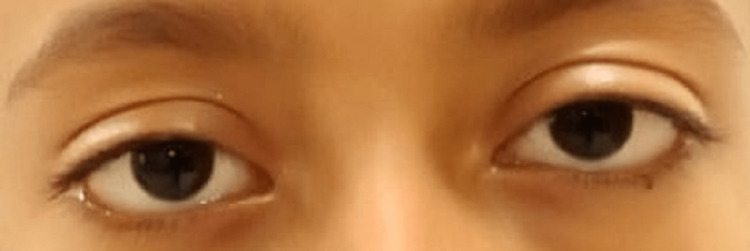
Preoperative frontal view of both eyes.

**Figure 2:**
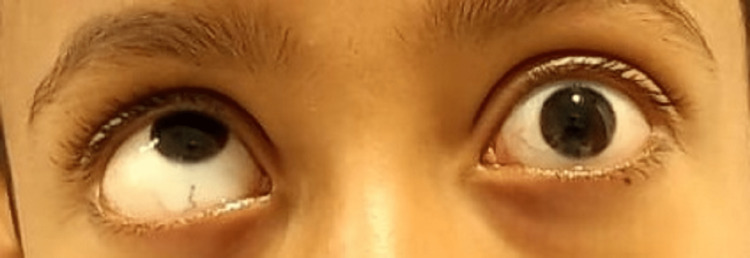
Preoperative view shows restricted movement in the left eye's upward gaze.

**Figure 3:**
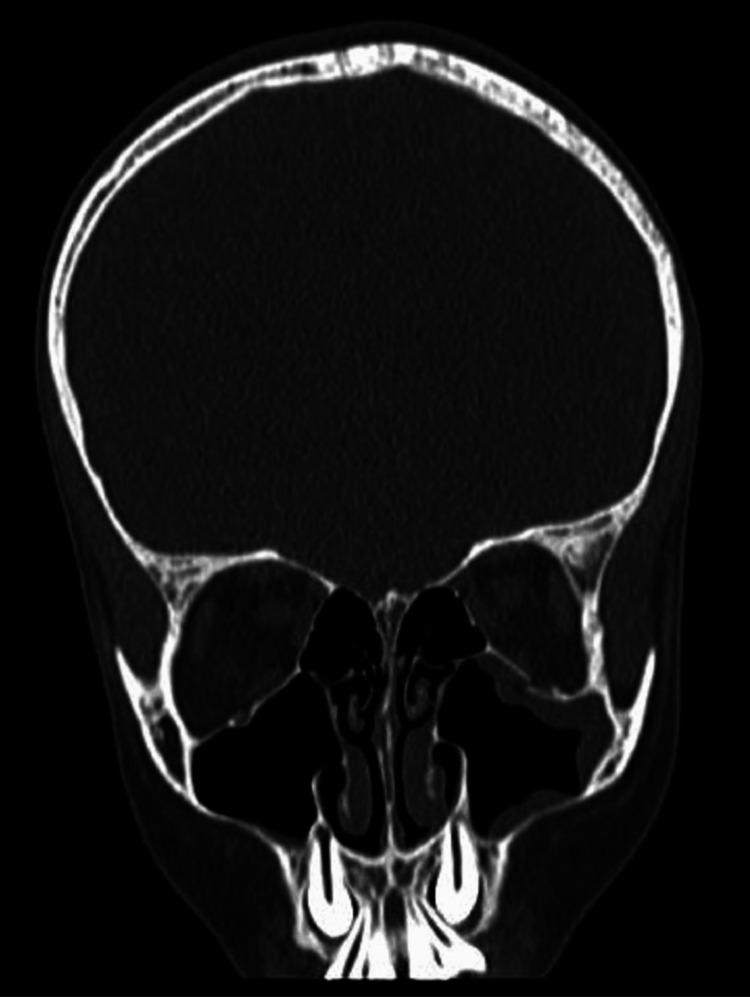
Coronal section of the patient's left eye CT orbit shows entrapment of the inferior rectus muscle. CT, computed tomography

A 10-year-old boy reported with his parents to the Pediatrics OPD in a tertiary care center in Sawangi, Wardha. The child complained of double vision in the left eye for seven days. The child gave a history of collision with another child while playing, following which he has an episode of vomiting and loss of consciousness. He was then taken to a nearby government hospital where a brain CT scan was done. The CT scan showed no evidence of intracranial injury, but there was evidence of floor-of-orbit fracture in the left eye. Upon receiving this diagnosis, the parents brought the child to a larger tertiary care center. The child was admitted to the Department of Pediatrics, where the further evaluation was done by an ophthalmologist and an oral surgeon. On examining both eyes, the extraocular muscle movements were found to be restricted in the left eye's upward gaze and the presence of binocular diplopia (Figures 1-2). The CT orbit findings confirmed the presence of a linear minimally displaced fracture of the floor of the left orbit (Figure 3). The patient was scheduled for surgery under general anesthesia. An intraoperative forced duction test was performed before starting the surgery. The test showed restriction in the left eye's upward movement. A transconjunctival incision was given in the left eye, and a lateral canthotomy was done.

**Figure 4:**
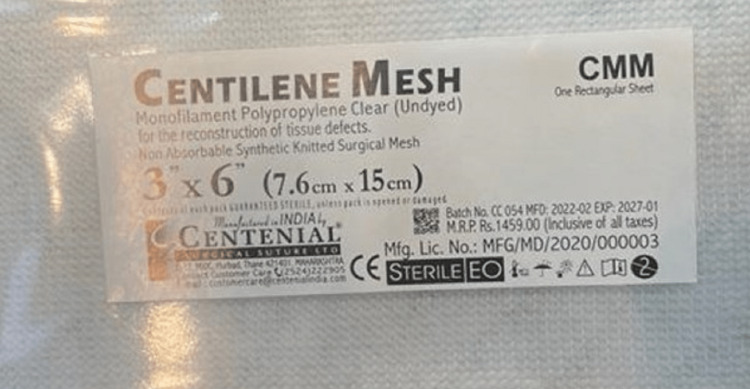
Centilene hernia mesh.

**Figure 5:**
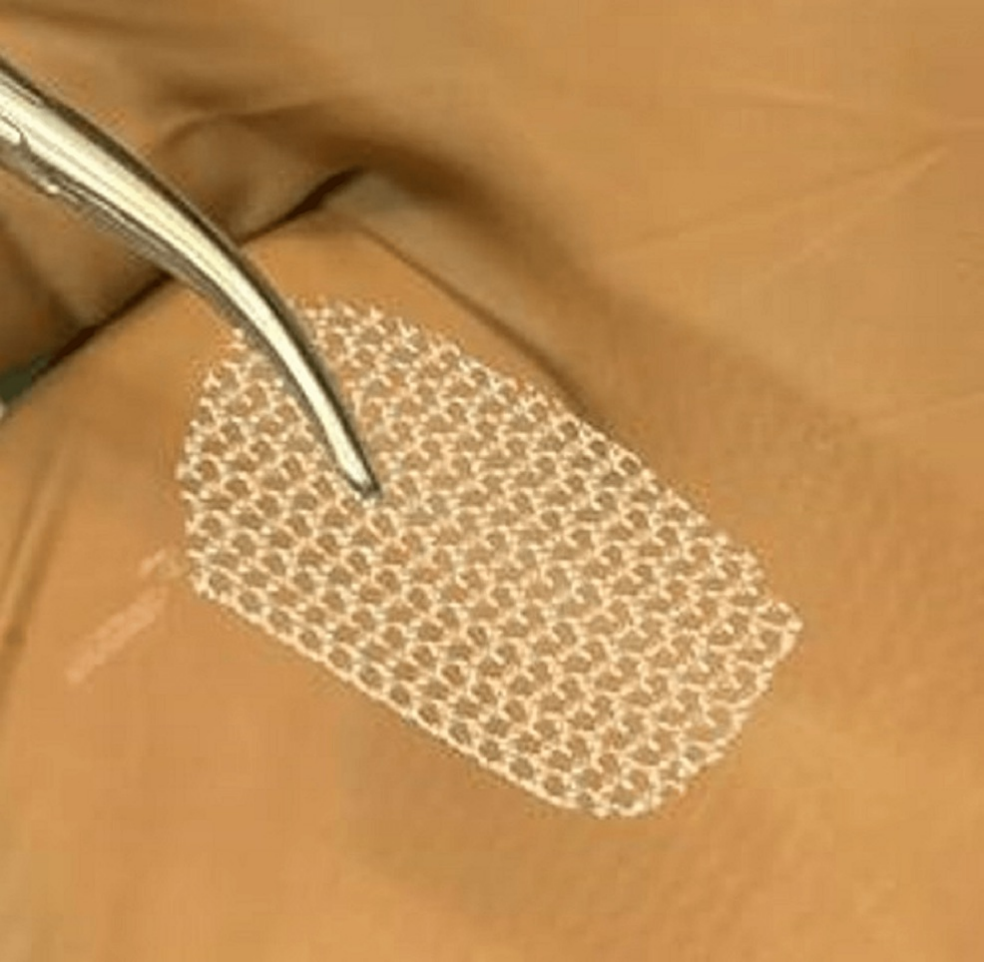
Centilene hernia mesh (15 mm × 10 mm × 0.55 mm).

**Figure 6:**
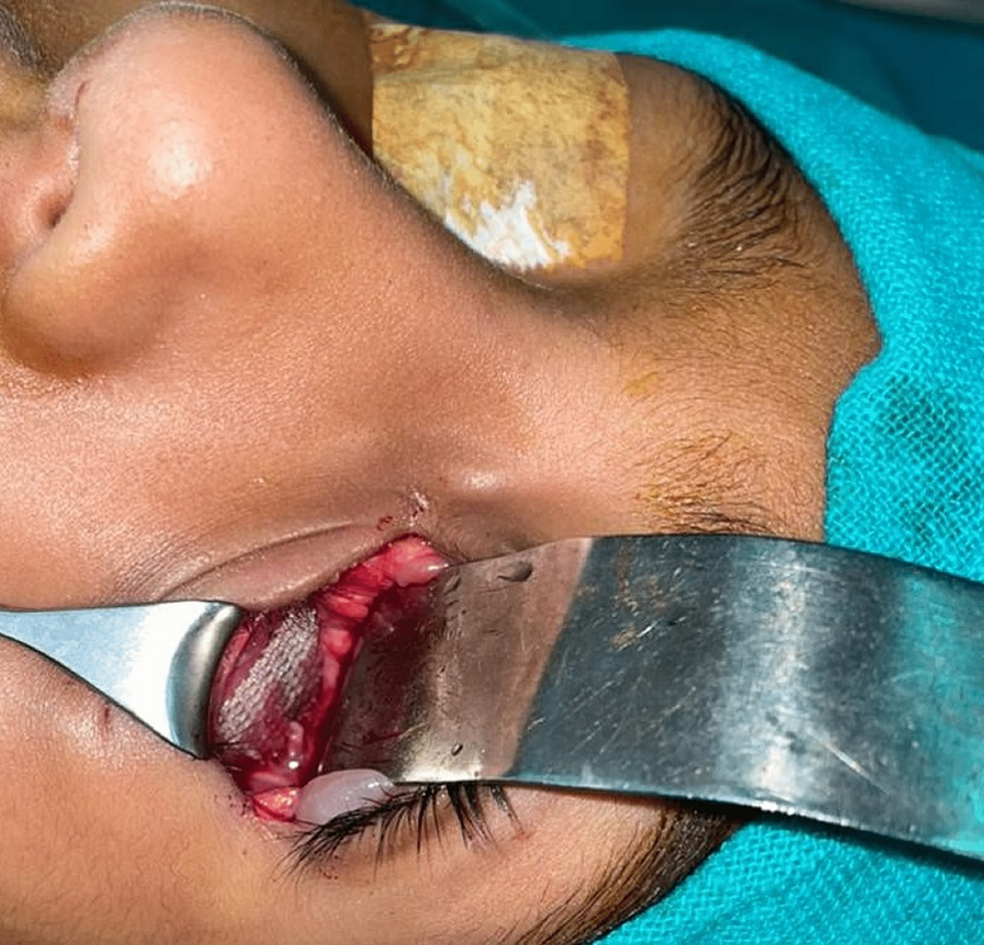
Intraoperative picture depicting the placement of nonresorbable polypropylene mesh on the floor of the left eye.

**Figure 7:**
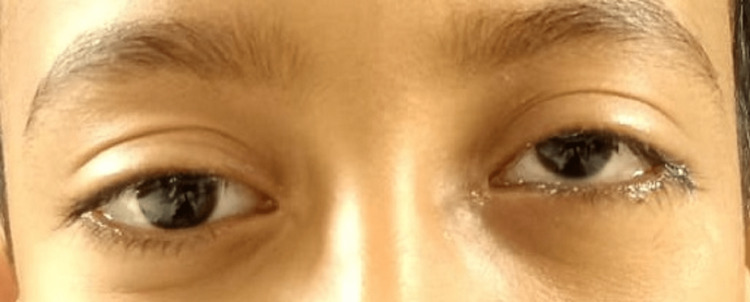
Postoperative frontal view of both eyes.

**Figure 8:**
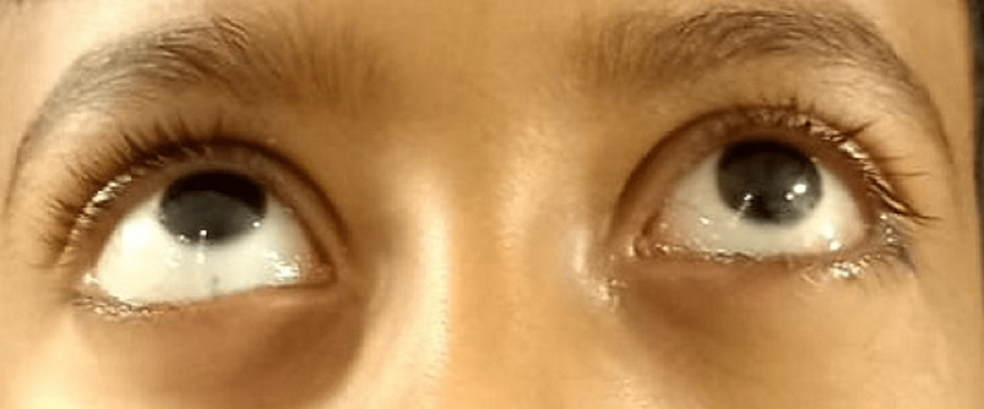
Marked improvement in the upward gaze movement at postop day 5.

The periorbital fat and tissues were retracted, and subperiosteal dissection was done until the fracture line was seen. The orbital defect reconstruction was first attempted with a traditional titanium orbital mesh. But the mesh could not be used as it was of unsuitable dimensions. A decision was then made to use a polypropylene hernia mesh instead of a titanium mesh. A monofilament nonresorbable polypropylene-based mesh (CENTENIAL, Thane, India) was used (Figure 4). The mesh was cut to the approximate dimensions of 15 mm × 10 mm × 0.55 mm (Figure 5) and placed over the orbital floor defect (Figure 6). The closure was done using a 5-0 Polyglactin Suture (CENTENIAL). The forced duction test was repeated. A simple dressing of cotton gauze was placed over the left eye. The patient was then reversed and shifted to the pediatric ICU for monitoring. The patient received intravenous (IV) Augmentin, fluids, and injectable dexamethasone (0.2 mg/kg) for three days. On postop day 5, there was a marked improvement in the upward gaze movement in the left eye (Figures 7-8). The diplopia resolved at a one-month follow-up with no restriction in the upward gaze.

## Discussion

Pediatric orbital fractures have a unique clinical presentation. The most common site of these fractures is the floor of the orbit, as reported by Kavak et al. [2]. They are associated with ocular findings, with the most common one being restriction in eye movements due to muscle entrapment [3]. Unlike orbital fractures in adults, orbital fractures in pediatric age groups present with few or no clinical features. However, *white-eyed* blowout fractures have also been reported in adults. In a retrospective case series, Ethunandan and Evans reported the occurrence of a *white-eyed* blowout fracture in an adult patient [6].

These categories of fractures seem to be associated with OCR, also known as the Aschner reflex or trigeminovagal reflex. The clinical features of this reflex are nausea, loss of consciousness, and bradycardia, which mimic the characteristics of head trauma. Due to the similarities between the two, orbital fractures are often misdiagnosed as head injuries. In a case report by Jurdy and Malhotra, they stated that the cases of orbital fractures, especially in the pediatric age group, could be underreported due to a misdiagnosis of intracranial injury [7].

Delay in diagnosing pediatric *white-eyed* blowout fractures can result in necrosis of entrapped muscle, permanent diplopia, loss of vision, and potentially fatal complications of trigeminocardiac reflex [8]. Correct diagnosis with prompt treatment is crucial for treating pediatric *white-eyed* blowout fractures. The risk of persistent diplopia is higher in the case of delayed surgical intervention [9]. There are several materials available for orbital reconstruction. Autologous materials such as iliac crest and nasal cartilage carry no risk of rejection and infection. They also provide good support. But they cause donor site morbidity and are difficult to shape. Allogenic materials also offer decent support, but they may cause infections. Alloplastic implants have gained popularity over the years, for reconstruction, especially for the small floor of the orbit defects. Alloplastic is easily available, and as reconstruction materials, they are reasonably priced and extremely easy to adapt [10]. Alkhalil and Otero found favorable results with minimum long-term complications in a case series of orbital repair using semiabsorbable polypropylene [5].

## Conclusions

This case report attempts to demonstrate numerous aspects of pediatric orbital fractures. These *white-eyed* blowout fractures of the orbit are frequently associated with the trigeminocardiac reflex. The signs of this reflex mimic those of head injury. Hence, these fractures are often misdiagnosed, and valuable time is wasted until the head injury diagnosis is ruled out. Orbital floor defect reconstruction using a nonresorbable synthetic mesh is not traditionally used in the pediatric age group. Further studies are needed to evaluate the effectiveness of nonresorbable polypropylene-based mesh in pediatric floor-of-orbit reconstructions.
